# Vaccination with human anti-trastuzumab anti-idiotype scFv reverses HER2 immunological tolerance and induces tumor immunity in MMTV.f.huHER2(Fo5) mice

**DOI:** 10.1186/bcr2826

**Published:** 2011-02-04

**Authors:** Maha Z Ladjemi, Thierry Chardes, Stephanie Corgnac, Veronique Garambois, Sebastien Morisseau, Bruno Robert, Caroline Bascoul-Mollevi, Imade Ait Arsa, William Jacot, Jean-Pierre Pouget, Andre Pelegrin, Isabelle Navarro-Teulon

**Affiliations:** 1 IRCM, Institut de Recherche en Cancérologie de Montpellier, INSERM U896, Université Montpellier1, CRLC Val d'Aurelle Paul Lamarque, 208 rue des Apothicaires, Montpellier, F-34298, France; 2 CRLC Val d'Aurelle-Paul Lamarque, 35 rue de la Croix Verte, Montpellier, F-34298, France

## Abstract

**Introduction:**

Novel adjuvant therapies are needed to prevent metastatic relapses in HER2-expressing breast cancer. Here, we tested whether trastuzumab-selected single-chain Fv (scFv) could be used to develop an anti-idiotype-based vaccine to inhibit growth of HER2-positive tumor cells *in vitro *and *in vivo *through induction of long-lasting HER-specific immunity.

**Methods:**

BALB/c mice were immunized with anti-trastuzumab anti-idiotype (anti-Id) scFv (scFv40 and scFv69), which mimic human HER2. Their sera were assessed for the presence of HER2-specific Ab1' antibodies and for their ability to reduce viability of SK-OV-3 cells, a HER2-positive cancer cell line, in nude mice. MMTV.f.huHER2(Fo5) transgenic mice were immunized with scFv40 and scFv69 and, then, growth inhibition of spontaneous HER2-positive mammary tumors, humoral response, antibody isotype as well as splenocyte secretion of IL2 and IFN-γ were evaluated.

**Results:**

Adoptively-transferred sera from BALB/c mice immunized with scFv40 and scFv69 contain anti-HER2 Ab1' antibodies that can efficiently inhibit growth of SK-OV-3 cell tumors in nude mice. Similarly, prophylactic vaccination with anti-Id scFv69 fully protects virgin or primiparous FVB-MMTV.f.huHER2(Fo5) females from developing spontaneous mammary tumors. Moreover, such vaccination elicits an anti-HER2 Ab1' immune response together with a scFv69-specific Th1 response with IL2 and IFN-γ cytokine secretion.

**Conclusions:**

Anti-trastuzumab anti-Id scFv69, used as a therapeutic or prophylactic vaccine, protects mice from developing HER2-positive mammary tumors by inducing both anti-HER2 Ab1' antibody production and an anti-HER2 Th2-dependent immune response. These results suggest that scFv69 could be used as an anti-Id-based vaccine for adjuvant therapy of patients with HER2-positive tumors to reverse immunological tolerance to HER2.

## Introduction

Breast cancer affects women worldwide and is a major public health problem. Despite progress in the field of surgery and adjuvant therapies, the risk of metastatic relapse remains high. Human Epidermal growth factor Receptor 2 (HER2) over-expression is observed in approximately 20% of invasive breast cancer and is an independent predictor of survival as it is associated with poor prognosis, aggressive disease and resistance to chemotherapy and hormone therapy [1-4]. HER2 has been targeted with immunotherapeutic approaches based on the use of anti-HER2 monoclonal antibodies (mAb), tyrosine kinase inhibitors and cancer vaccines [5].

Patients with HER2-expressing tumors show HER2-specific humoral and/or T-cell responses [6,7]. Such anti-HER2 immune responses, albeit of low magnitude, indicate that HER2 is a suitable candidate for HER2-targeted vaccine strategies. Induction of a stronger HER2-specific immunity with anti-tumor vaccines should lead to the establishment of immune memory, thereby preventing tumor recurrence, metastasis and relapse. However, HER2-induced immunological tolerance has been described, probably related to its oncofetal origin, which is an obstacle to efficient vaccination against this antigen [8]. To circumvent self antigen-dependent tolerance, peptide-, DNA- or anti-Idiotype (Id)-based vaccines have been developed that show great specificity without notable toxicity [9-14]. Among them, anti-Id antibodies have been proposed as vaccines for cancer immunotherapy and significant success has been achieved using anti-Id vaccines mimicking tumor-associated antigens (TAAs). This approach is based on N.K. Jerne's idiotype network theory about the Ab1-Ab2-Ab3 antibody cascade stimulation, whereby specific anti-Id antibodies (Ab2β induced by immunization with antigen-specific Ab1 antibodies, can serve as an "internal image" of the target antigen and can be used to induce Ab3 (also named Ab1') antibodies that can bind to the cognate antigen [15]. Previous studies have described the use in solid tumors of anti-Id mAbs, which mimic TAAs, such as carcinoembryonic antigen (CEA), disialoganglioside GD2 or cancer-antigen 125 (CA-125), and demonstrated that these anti-Id mAbs induce an antigen-specific humoral response [16-19]. In clinical trials, including patients with ovarian carcinoma, colorectal carcinoma or malignant melanoma, anti-Id-specific humoral and/or cellular responses following immunization were associated with a better survival rate without toxicity, but with modest objective responses. Available results of treatment of breast cancer patients with anti-Id mAbs are still very preliminary and conclusions go no further than the mere biologic proof of principle [20].

In this context, our goal was to develop a vaccine to boost anti-HER2 immunity in patients with HER2-positive tumors and pre-existent low-level immunity. To this end, the use of HER2-mimicking anti-Id antibodies as a vaccine is a promising alternative. In a previous work [21], we reported that two human anti-Id scFv antibody fragments (scFv40 and scFv69), which were selected by screening a phage-displayed library using the anti-HER2 antibody trastuzumab, induced an anti-HER2 antibody response in sera of immunized BALB/c mice. In the present study, we show that immunization with anti-Id scFv40 and scFv69 induces production of Ab1' that inhibit growth of HER2-positive tumor cells both *in vitro *and *in vivo*. Moreover, prophylactic vaccination with anti-Id scFv69 efficiently protects MMTV.f.huHER2(Fo5) mice from developing spontaneous HER2 positive mammary tumors through the induction of a HER2-specific Ab1' antibody response. Taken together, these results indicate that the anti-Id scFv69 fragment could be envisaged as an anti-idiotype-based vaccine for adjuvant therapy in patients with HER2-positive tumors, through reversion of HER2-specific immunological tolerance.

## Materials and methods

### Reagents

The recombinant HER2-Fc fusion protein (kindly provided by Pr. J.P. Mach, Biochemistry Institute, University of Lausanne, Switzerland) is composed of two extracellular domains of human HER2 linked with a human Fc fragment and was produced by transfection of Human Embryonic Kidney 293 cells. The anti-HER2 humanized antibody trastuzumab (Herceptin^®^) was purchased from Genentech, Inc. (San Francisco, CA, USA). Production, purification and characterization of anti-Id trastuzumab-selected scFv40 and scFv69 have already been described [21]. The irrelevant scFv 13R4 is a kind gift from Dr. P. Martineau (IRCM, Montpellier, France). MFE23-Fc fusion protein is a kind gift from R. Kontermann (University of Stuttgart, Germany).

### Cell lines

The HER2-overexpressing human ovarian carcinoma cell line SK-OV-3 and the Chinese hamster ovarian (CHO) cell line, were obtained from the American Type Culture Collection (ATCC; Rockville, MD, USA). SK-OV-3 cells were cultured in DMEM medium (Gibco, Paisley, UK) and CHO cells in RPMI-1640 medium (Gibco), both supplemented as recommended by ATCC. Cells were maintained at 37°C in a humidified atmosphere with 5% CO_2_.

### Mice

All *in vivo *experiments were performed in compliance with the French guidelines for experimental animal studies (Agreement N° B34-172-27). Six-to eight-week-old female BALB/c, FVB (the mouse strain used to generate the MMTV.f.huHER2(Fo5) transgenic line) and nude athymic nude mice were purchased from Harlan Laboratories (Germany). The transgenic mouse line MMTV.f.huHER2(Fo5) was obtained from Genentech and has been previously described [22]. These mice, which over-express human HER2 under the control of the MMTV promoter and spontaneously develop mammary tumors within an average time of seven months from birth, are used as a pre-clinical model of HER2-overexpressing breast cancer. They were maintained in the IRCM animal facilities and used for immunization when they were three-month-old. For *in vivo *experiments, body weight was assessed weekly, as a surrogate marker of toxicity for the duration of the experiments, concomitantly with tumor measurement.

### Anti-Id scFv mouse immunization

Immunogens were first injected subcutaneously (s.c.) after emulsion with Complete Freund Adjuvant (CFA) in BALB/c, FVB or MMTV.f.huHER2(Fo5) mice, followed two weeks later by a second s.c. administration with Incomplete Freund Adjuvant (IFA). Then, two intraperitoneal (i.p.) injections with IFA were given at day 21 and 35 after the initial boost. In FVB-MMTV.f.huHER2(Fo5) mice, a final i.p. injection with IFA was given at Day 90 after the initial boost. Immunogens were anti-Id scFv40 and scFv69 (50 μg/inj), HER2-Fc fusion protein (20 μg/inj), or phosphate-buffered saline PBS (negative control) at a 1:1 ratio with CFA or IFA. For serum antibody measurements, mice were bled and sera were drawn from the tail vein at various times during the experiment and stored at -20°C until assay.

### ELISA and flow cytometry analysis of HER2-specific Ab1' antibodies in sera of vaccinated mice

Indirect ELISA and flow cytometry analysis were performed, as previously described [21], to detect the presence of anti-HER2 antibodies (Ab1') in sera of immunized mice. Briefly, indirect ELISA was performed by coating 96-well plates overnight at 4°C with HER2-Fc fusion protein diluted at 5 μg.ml^-1 ^in PBS and then saturated for one hour with PBS containing 1% serum albumin (BSA). After four washings with 0.1% Tween/PBS, 100 μl of diluted sera (1:50) from immunized mice were added to each well and incubated for 1.5 h at room temperature. Secondary goat anti-mouse IgG (γ-chain specific)-horseradish conjugate (Millipore Chemicon, Billerica, MA, USA) was added (dilution 1:2,500) after plate washing, incubated for 1.5 h, and then *ο*-phenylenediamine substrate was added for revelation according to the supplier's instructions (Sigma, St Louis, MO, USA). After stopping the reaction with 50 μl of 3N HCl/well, absorbance was measured at 490 nm, using an ELISA microreader (Multiskan EX, Thermo Electron Corporation, Vantaa, Finland).

For flow cytometry analysis, SK-OV-3 and CHO cells were incubated for one hour at 4°C with 100 μl of each mouse serum (diluted at 1:50) or 100 μl of trastuzumab (at 20 μg/ml) diluted in PBS containing 10% fetal calf serum. After washing, cells were incubated at 4°C with either 100 μl of sheep anti-mouse IgG-FITC-labeled antibody (1:100; Sigma-Aldrich, Saint Louis, MO, USA) for sera or anti-human-FITC-labeled antibody (1:100; Sigma-Aldrich) for trastuzumab for 45 minutes. Cells were then suspended in 500 μl PBS and 10,000 events were analyzed with a FACScan apparatus (Becton Dickinson, Franklin Lakes, NJ, USA).

### Stable transfection of SK-OV-3 cells with luciferase

To image tumor cells in nude mice, SK-OV-3 cells were transfected with a luciferase-expressing vector (CMV-Luc-Hygro), a gift from Dr. P. Balaguer (U896, Institut de Recherche en Cancérologie de Montpellier, France), using a Calcium Phosphate transfection kit (Sigma). Transfected cells (SK-OV-3-Luc) were selected with 400 μg/ml Hygromycin and individual clones were analyzed for luciferase expression by measuring luciferase activity of cells plated in 96 well/plates with a luminometer (Microbeta, Perkin-Elmer Wallac, Waltham, MA, USA). HER2 expression in SK-OV-3-Luc clones was measured by flow cytometry with a FACScan flow cytometer (Becton Dickinson, San Jose, CA, USA).

### Cell viability

Sera from immunized BALB/c mice were harvested at Day 42 after the initial boost, pooled, sterile-filtered and stored at -20°C until use. HER2-specific antibodies were detected in the serum as described above. A total of 1 × 10^4 ^SK-OV-3-Luc cells/well were plated in 96-well plates in 100 μl of culture medium and grown at 37°C for 24 h. Thereafter, 100 μl of a 1:5 dilution of sera from immunized BALB/c mice were added to the culture medium. On Day 5, 50 μl of luciferin solution (Promega), at a final concentration of 3 × 10^-4 ^M, were added to the cells and luminescence was measured with a luminometer (Microbeta, Wallac). Cell number was proportional to the emitted luminescence. The results were normalized to the luminescence of non-treated cells. Each condition was done in triplicate in two independent experiments.

### Adoptive transfer of immune sera from anti-Id scFv-vaccinated mice

Female athymic nude mice were i.p. injected with 5 × 10^5 ^SK-OV-3-Luc cells/mouse and grouped (*n *= 5 animals/group) at Day 3 after injection. At Day 4 post-graft, mice were i.p. injected with 200 μl of mouse serum from naïve donors, or from BALB/c mice immunized with anti-Id scFv40, anti-Id scFv69, or HER2-Fc once a week for four weeks. Trastuzumab (200 μg/inj) was used as a positive control of the experiment. Since the tumor size is proportional to the emitted luminescence, the efficiency of the different treatments was evaluated by measuring the luminescence once a week following i.p. injection of luciferin (100 μg/g). Mice were sacrificed when tumors reached the luminescence level of 3 × 10^7 ^photon/second. The results were expressed by an adapted Kaplan-Meier survival curve, using the time taken for a tumor to reach this level of emitted luminescence. Moreover, a median delay was defined as the time needed to 50% of the mice to reach that luminescence.

### *In vivo *prophylactic vaccination with anti-Id scFv40 or anti-Id scFv69

MMTV.f.huHER2(Fo5) and FVB mice were immunized following the vaccination schedule described above when they were three months old. Follow-up of spontaneous development of mammary tumors was performed by direct palpation of the mammary glands for the entire duration of the experiment. Results were expressed by an adapted Kaplan-Meier curve of tumor-free survival using the age of palpable tumor onset in each mice. Moreover, the median delay was defined as the age when 50% of the mice had developed palpable mammary tumors. The study was ended at 59 weeks of age.

### *In vitro *stimulation of splenocytes and analysis of IL2 and IFNγ production in culture supernatants

Six days after the final boost, the spleen of one immunized MMTV.f.huHER2(Fo5) mouse from each group was excised and pressed through stainless mesh. Isolated cells were washed with RPMI medium and then erythrocytes were lysed by hypotonic shock with 0.83% ammonium chloride solution. Splenocytes (5 × 10^5 ^per well) were seeded in flat-bottomed 96-well culture plates in triplicates in the presence of scFv69 and HER2-Fc (2.0 μg/ml/each) as stimulating agents. Mfe23-Fc fusion protein and scFv13R4 (2.0 μg/ml/each) were used as irrelevant proteins and 7.5 μg/ml Concanavalin A was used as a positive control. Cell-free supernatants were recovered 24 h and 72 h post-stimulation and were analyzed for IL2 and IFN-γ production by using the ELISA kit (BD Biosciences, San José, CA, USA) according to the manufacturer's instructions.

### Immunoglobulin isotyping

Isotype distribution of anti-scFv69- and -HER2-Fc specific antibodies generated in immunized MMTV.f.huHER2(Fo5) and FVB mice was determined using a mouse monoclonal isotyping kit (Pierce, Rockford, IL, USA) according to the manufacturer's instructions.

### Statistical methods

Concerning the *in vitro *assays, comparison of the percentage of cell viability inhibition between the different groups and normal mouse sera (NoMS) was performed with the Wilcoxon two-sample exact test for all continuous variables. The HER2-specific humoral responses in mice immunized with PBS or with scFv69 were compared with the Wilcoxon two-sample exact test. Differences were considered statistically significant when *P *< 0.05. Survival rates were estimated from the time of the xenograft until the date of the event of interest using the Kaplan-Meier method. Median survival was presented with 95% confidence intervals. The event of interest was a bioluminescence value of 3 × 10^7 ^photon/second for athymic nude mice and the appearance of palpable mammary tumors for huHER2-transgenic mice. Survival curves were compared using the Log-rank test. Statistical analysis was performed using the STATA 10.0 software (StataCorp LP, College Station, TX, USA).

## Results

### Anti-Id immunization of BALB/c mice with scFv40 or scFv69 induces anti-HER2 humoral Ab1' immune response

**Figure 1:**
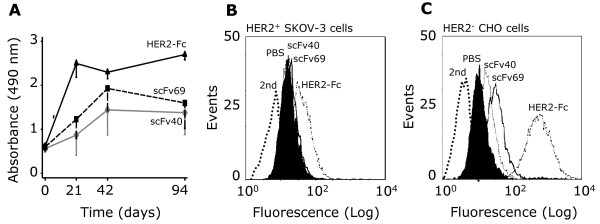
**Anti-HER2 antibodies are present in sera from BALB/c mice immunized with anti-Id scFv40 and scFv69**. **(A) **ELISA of pooled sera from mice (*n *= 20/group) immunized with scFv40, scFv69 or HER2-Fc. Values are presented as mean ± SD of the Absorbance at 490 nm from three independent experiments. Flow cytometry analysis in **(B) **HER2-overexpressing SK-OV-3 and **(C) **HER2-negative CHO cells of the HER2-specific response of pooled sera from mice immunized with PBS, anti-Id scFv40, scFv69 or with HER2-Fc. Sera were recovered at Day 42 post-vaccination. Antibody binding was detected using a FITC-labeled goat anti-mouse antibody. FITC-labeled secondary antibody alone was used as a negative control to determine the level of background fluorescence.

To test whether the trastuzumab-specific anti-Id scFv40 and scFv69 we previously isolated could be used to develop a vaccine to boost anti-HER2 immunity, we immunized six- to eight-week-old BALB/c mice (*n *= 20 animals/group) with scFv40, scFv69, HER2-Fc fusion protein, or PBS (negative control). HER2-specific antibodies were detected by ELISA in pooled sera from the different groups of immunized mice (Figure 1A). Specifically, scFv-induced anti-HER2-Ab1' antibodies were evidenced at 21 days post-immunization, increased until Day 42 post-immunization, and persisted throughout the experiment (Day 94). As expected, anti-HER2 antibody titers were higher in sera from animals immunized with HER2-Fc (HER2-FcMS) than in sera from mice immunized with scFv40 (scFv40MS) and scFv69 (scFv69MS). This was partially due to an anti-Fc response, which corresponded to approximately 30% of the whole antibody response in HER2-FcMS, as previously demonstrated by ELISA using an irrelevant Fc-bearing fusion protein [21]. Nevertheless, in all three groups the antibody titers were significantly higher at Day 94 post-immunization than at Day 0 as indicated by the Student's *t-*test analysis of the mean values of Absorbance between the two time points (*P *= 0.049 for scFv40, *P *= 0.042 for scFv69 and *P *< 0.001 for HER2-Fc). The specificity of the Ab1' response in scFv40MS and scFv69MS was confirmed by flow cytometry using HER2-positive SK-OV-3 and HER2-negative CHO cells. Binding to HER2 was detected in SK-OV-3 cells incubated with scFv40MS, scFv69MS or HER2-FcMS (Figure 1B), but not with sera of mice immunized with PBS (NoMS). Binding was not observed following incubation of CHO cells with any of the tested sera (Figure 1C). These results demonstrate that in mice, anti-idiotype immunization with trastuzumab-specific scFv induces an anti-anti-Id Ab1' humoral response against the HER2 cognate antigen.

### Sera from mice immunized with scFv40 and scFv69 reduce viability of SK-OV-3 cells

**Figure 2:**
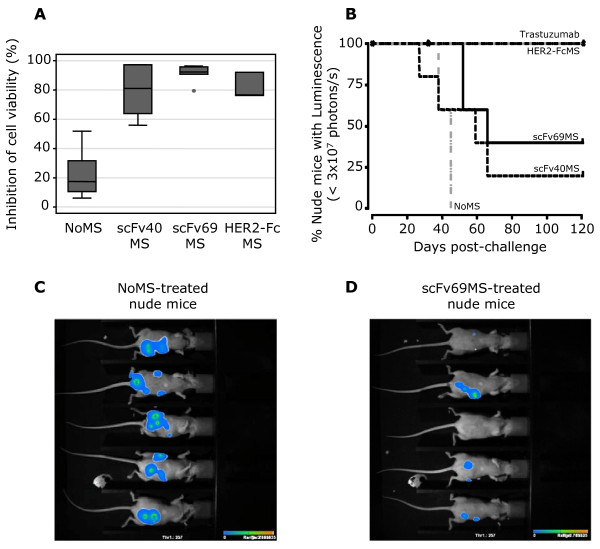
**Inhibition of cell viability and analysis of SK-OV-3 tumor progression in nude mice**. **(A) **Inhibition of cell viability in SK-OV-3 Luc cells following treatment with sera from BALB/c mice immunized with PBS (NoMS), scFv40 (scFv40MS), scFv69 (scFv69MS) or with HER2-Fc (HER2-FcMS). Cell number was proportional to the emitted luminescence and results were normalized to the fluorescence of untreated cells. Grey boxes represent the 25^th ^and 75^th ^percentiles with the medians as black lines; whiskers mark the smallest and largest non-outlier observations, and outliers are indicated by dots. **(B) **Analysis of tumor progression in nude mice (*n *= 5/group) xenografted with HER2-overexpressing SK-OV-3-Luc tumor cells and then adoptively transferred by i.p. injection of 200 μl of scFv40MS, scFv69MS, HER2-FcMS or NoMS (negative control), diluted 1:5, at Day 4 after xenograft. Positive controls were animals treated with trastuzumab (200 μg/injection). Tumor growth was evaluated by measuring the emitted luminescence once a week following injection of luciferin. Bioluminescence imaging of nude mice xenografted with SK-OV-3-Luc tumor cells and treated with NoMS **(C) **or scFv69MS **(D) **was performed at the end of the treatment (Day 27 after xenograft).

We then evaluated the biological activity of scFv40 and scFv69 sera by assessing their ability to inhibit cell growth *in vitro*. To this end, SK-OV-3 cells were stably transfected with a luciferase-expressing vector (CMV-Luc-Hygro) and clone D8 was selected by limiting dilution for its high HER2 expression combined with luciferase activity (SK-OV-3-Luc cells). SK-OV-3-Luc cells were then incubated with a 1:5 dilution of scFv40MS, scFv69MS, HER2-FcMS and NoMS for five days and cell viability was assessed based on the level of luciferase activity. Results were presented as the percentage of inhibition of cell viability. Cell growth was efficiently inhibited by scFv40MS (81% mean inhibition), scFv69MS (92.4%) and HER2-FcMS (76.6%) but not by NoMS (17.5%, *P *= 0.002 NoMS *vs *scFv) (Figure 2A). These results demonstrate that HER2-specific Ab1' antibodies from sera of anti-Id scFv-immunized mice can inhibit up to 92% of cell growth in a HER2-overexpressing human cancer cell line *in vitro*.

### Adoptive transfer of sera from mice immunized with scFv40 or scFv69 inhibits tumor growth in athymic nude mice

To evaluate *in vivo *the therapeutic efficiency of the anti-HER2 Ab1' antibodies, we injected scFv40MS, scFv69MS or HER2-FcMS, NoMS (negative control) and trastuzumab (anti-HER2 antibody, positive control) in athymic nude mice (*n *= 5 for each group), which had been grafted four days before with HER2-positive SK-OV-3-Luc tumor cells. Tumor growth was evaluated weekly in each group by measuring the luminescence emitted following luciferin injection. As expected, all mice treated with trastuzumab or with HER2-FcMS were cured (that is, luminescence in the surviving animals was below the threshold level of 3 × 10^7 ^photons/s) at the end of the experiment (Day 120), whereas mice treated with NoMS were all dead by Day 45 post-challenge (Figure 2B). Mice treated with scFv sera had a survival rate of 20% (remission in one out five mice with scFv40MS) and 40% (remission in two out of five mice with scFv69MS) at the end of the experiment (Day 120). Overall, the median delay (Figure 2B) for tumors to reach the luminescence level of 3 × 10^7 ^photon/second was significantly longer in mice treated with scFv69MS (66 days) than in mice treated with NoMS (45 days, *P *= 0.0031). The higher survival rate of mice treated with HER2-FcMS is in accordance with the highest titer of anti-HER2 antibodies in HER2-FcMS than in scFv sera. Bioluminescence analysis of xenografted nude mice at Day 27 after challenge (corresponding to two days after the last treatment) demonstrated smaller luminescence patches in mice treated with scFv69MS (Figure 2D) than in those treated with NoMS (Figure 2C). Moreover, the two mice in remission after treatment with scFv69MS did not present detectable bioluminescence patches. These data clearly demonstrate the efficiency of the HER2-specific Ab1' antibodies from sera of anti-Id scFv-immunized mice to *in vivo *inhibit growth of SK-OV-3 HER2-positive cancer cells. Altogether, these *in vitro *and *in vivo *evidences gave us the proof of concept that vaccination with trastuzumab-specific anti-Id scFv40 or scFv69 generates HER2-specific Ab1' antibodies. When adoptively-transferred, these antibodies inhibit tumor growth in athymic nude mice xenografted with HER2-overexpressing cancer cells.

### Prophylactic vaccination of MMTV.f.huHER2(Fo5) transgenic mice with anti-Id scFv69 induces an anti-HER2 Ab1' immune response followed by tumor inhibition

**Figure 3:**
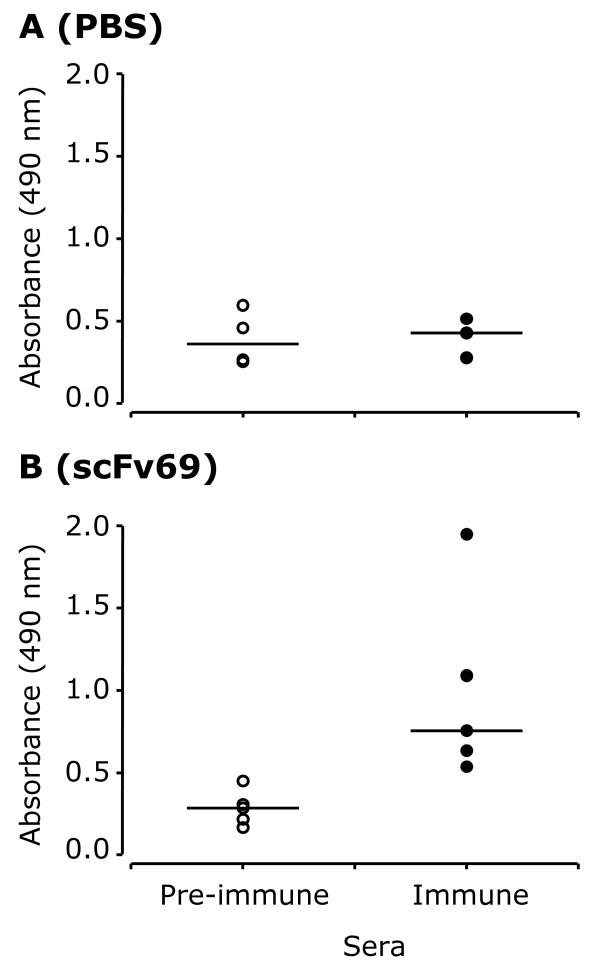
**Analysis of the HER2-specific antibody response in sera of MMTV.f.huHER2(Fo5) transgenic mice**. Mice were immunized with PBS (*n *= 4) **(A) **or anti-Id scFv69 (*n *= 5) **(B)**. HER2-specific antibody level was measured in sera collected before the first immunization (pre-immune sera) and at the time of the maximum response during the study (Day 42) (immune sera). Each dot represents an immunized mouse.

**Figure 4:**
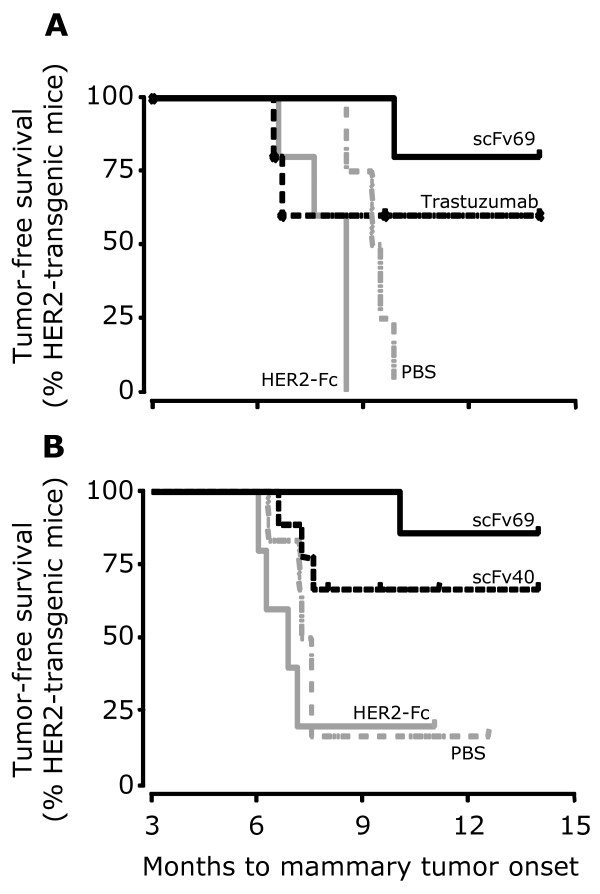
***In vivo *prophylactic vaccination with anti-Id scFv protects against spontaneous mammary tumor development in MMTV.f.huHER2(Fo5) transgenic mice**. The Kaplan-Meier disease-free survival plots illustrate the age of appearance of mammary tumors in **(A) **virgin females vaccinated with PBS (negative control), anti-HER2 trastuzumab, scFv69 or HER2-Fc and **(B) **primiparous females vaccinated with PBS (negative control), anti-Id scFv40 or scFv69, and HER2-Fc.

To further confirm the potential benefits of vaccination by anti-Id scFv69, we performed a prophylactic vaccination experiment using the MMTV.f.huHER2(Fo5) transgenic mouse model [22]. This pre-clinical mouse model, which is characterized by HER2 over-expression and spontaneous development of mammary cancer in females, has been validated for the study of targeted anti-HER2 therapies such as trastuzumab [22]. Furthermore, we have previously determined that MMTV.f.huHER2(Fo5) transgenic mice are tolerant for the human HER2 antigen by showing that, contrary to wild type mice, they were unable to mount a specific immunological response against HER2 following vaccination with recombinant HER2-Fc. In the present study, again we used MMTV.f.huHER2(Fo5) mice to evaluate the anti-HER2 response induced by immunization with PBS alone, anti-Id scFv69 or HER2-Fc. A HER2-specific humoral response was detected only in sera of mice immunized with scFv69 (*n *= 5, *P *= 0.016) (Figure 3B), but not in sera of animals immunized with PBS (*n *= 4) (Figure 3A) or with HER2-Fc (*n *= 5). The antibodies elicited in MMTV.f.huHER2(Fo5) mice immunized with HER2-Fc were strictly directed against the human Fc portion of the recombinant protein (data not shown). Furthermore, the survival curves (Figure 4A), revealed that spontaneous mammary tumors occurred in all mice immunized with PBS or HER2-Fc, confirming that MMTV.f.huHER2(Fo5) mice are tolerant for the human HER2 antigen. Conversely, all but one of the MMTV.f.huHER2(Fo5) mice immunized with anti-Id scFv69 were protected from developing HER2-positive mammary tumors throughout the experiment (14 months) (survival rate = 80%) (Figure 4A). Five additional animals were treated with trastuzumab (200 μg/inj) (positive control). Two out of five developed spontaneous mammary tumors, in accordance with the results previously described by Finkle *et al*., indicating that about 60% of mice treated with trastuzumab did not present clinical symptoms [22]. These results suggest a significant disease-free survival advantage for MMTV.f.huHER2(Fo5) mice vaccinated with anti-Id scFv69 in comparison to PBS-immunized (*P *= 0.0027) and HER2-Fc-immunized mice (*P *= 0.0034).

To check whether anti-Id scFv vaccination could protect MMTV.f.huHER2(Fo5) females from developing mammary tumors after the physiological changes linked to pregnancy, we carried out a second experiment in which females were immunized with PBS alone (*n *= 6), scFv40 (*n *= 9), scFv69 (*n *= 7) or HER2-Fc (*n *= 6) according to the usual protocol and then underwent pregnancy. Protection against spontaneous mammary tumors was observed in six (about 67%) out of nine of the scFv40- and six (about 85%) out of seven of the scFv69-vaccinated transgenic females, whereas PBS-immunized or HER2-Fc-immunized mice were not protected (Figure 4B). Taken together, these results demonstrate that prophylactic vaccination of MMTV.f.huHER2(Fo5) transgenic mice with anti-Id scFv69 (and to a lesser extent with scFv40) induces an anti-HER2 Ab1' immune response and protects mice from developing spontaneous HER2-positive tumors. Thus, this scFv fragment could be considered as a potential anti-Id based vaccine for adjuvant therapy of patients bearing HER2-positive tumors.

### Prophylactic vaccination of MMTV.f.huHER2(Fo5) transgenic mice with anti-Id scFv69 induces scFv69-specific Th1 and HER2-specific Th2 immune responses

**Figure 5:**
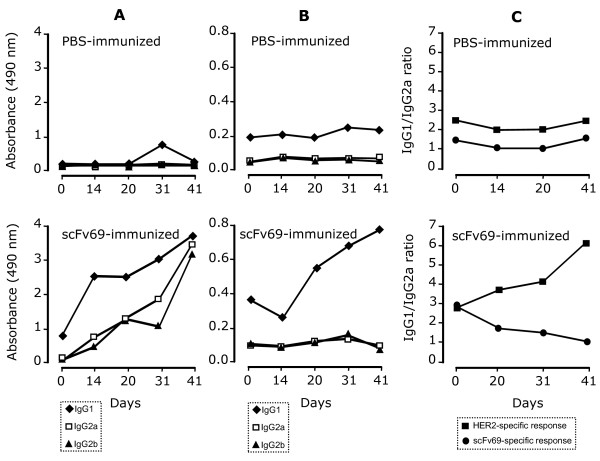
**Analysis of the immune responses induced by vaccination with anti-Id scFv69**. IgG1, IgG2a and IgG2b isotype distribution of anti-scFv69 **(A) **and anti-HER2 **(B) **antibodies in sera from MMTV.f.huHER2(Fo5) transgenic mice immunized with PBS (upper panels) or scFv69 (lower panels); **(C) **IgG1/IgG2a ratio of the anti-scFv69 or anti-HER2 response in transgenic mice immunized with PBS or scFv69.

To better characterize the immune response elicited by the prophylactic vaccination, splenocytes were isolated from PBS- or scFv69-immunized MMTV.f.huHER2(Fo5) mice, six days after the final boost (Day 41). Stimulation of spleen with the mitogen Concanavalin A (positive control) induced secretion of IL2 and IFNγ in supernatants of both PBS- and scFv69-immune splenocytes (Table 1). The faster secretion kinetics of IL2 (24 h) and the intermediate kinetics of IFN-gamma (48 h) are in agreement with the kinetics observed upon activation of T helper cell populations. Stimulation with scFv69 induced secretion of IL2 and IFN-gamma at comparable levels only in splenocytes isolated from mice vaccinated with anti-Id scFv69. Stimulation with HER2-Fc or the irrelevant Mfe23-Fc and scFv13R4 did not induce any detectable or significantly different secretion of IL2 or IFNγ (Table 1). The weak stimulation observed with the control scFv13R4 could be due to the presence of cell wall-derived lipopolysaccharides, which have the capacity to induce cytokines (Table 1). To further typify the Th1 and Th2 immune responses induced by vaccination with scFv69, we determined the scFv69- and HER2-specific antibody isotypes and the IgG1/IgG2a ratio by ELISA in sera from PBS- and scFv69-immunized MMTV.f.huHER2(Fo5) mice (Figure 5). Whereas no significant antibody response, regardless of the isotype, was observed in sera from mice immunized with PBS (upper panels of Figure 5), a progressively increasing anti-scFv69 response of IgG1, IgG2a and IgG2b isotype was observed until Day 41 (end of experiment) in sera from mice immunized with scFv69 (Figure 5A lower panel). In contrast, the HER2-specific response started only at Day 14 until the end of the experiment and was exclusively of the IgG1 isotype (Figure 5B lower panel). The scFv69-specific IgG1/IgG2a ratio progressively decreased during the vaccination schedule, thus confirming that, together with IFNγ/IL2 secretion, scFv69 prophylactic vaccination of MMTV.f.huHER2(Fo5) transgenic mice induced a scFv69-specific Th1-type immune response. Conversely, the anti-HER2 IgG1/IgG2a ratio increased over time (Figure 5C lower panel), demonstrating that prophylactic vaccination of MMTV.f.huHER2(Fo5) transgenic mice with scFV69 induced a HER2-specific Th2-type immune response.

**Table 1:** Cytokine secretion in antigen-stimpulated splenocytes from MMTV.f.huHER2(Fo5) transgenic mice immunized with anti-idiotype scFv 69 vaccine

Cytokine	Conc	HER2-transgenic mouse immunized with	
Stimulating antigen	(μg/ml)		
		
		PBS	scFv69
**Interleukin-2**			
Con A	7.5	356.9 ± 5.7	422.8 ± 26.5
Control scFv 13R4	2.0	<4	<4
ScFv69	2.0	<4	10.6 ± 0.8
Control Mfe23-Fc	2.0	<4	<4
HER2-Fc	2.0	<4	<4
			
**Interferon-gamma**			
Con A	7.5	21.4 ± 0.9	24.2 ± 0.8
Control scFv 13R4	2.0	<1	7.0 ± 3.6
ScFv69	2.0	<1	17.0 ± 3.7
Control Mfe23-Fc	2.0	<1	<1
HER2-Fc	2.0	<1	<1

*ng/ml.

Cytokine (Il-2 and IFN-gamma) secretion in splenocytes from MMTV.f.huHER2(Fo5) transgenic mice immunized with PBS or anti-Id scFv69. Splenocytes were isolated on Day 42 post-immunization. Supernatants from antigen-stimulated cells were recovered 24 h (for IL-2) and 72 h (for IFN-gamma) after *in vitro *stimulation. IL-2 and IFN-gamma detection (ng/ml) were performed using an ELISA assay, with a standard curve obtained with recombinant IL-2 (detection limit 4 ng/ml) or recombinant IFN-gamma (detection limit 1 ng/ml). Results are the mean ± SD of triplicate wells and are representative of two independent experiments. Mfe23-Fc and scFv13R4 are two irrelevant antigens. Con A, Concanavalin A.

## Discussion

Several immunotherapeutic approaches targeting HER2 have been reported in the literature, including both passive and active immunization [23]. The major problem in the use of HER2 as a target for active immunotherapy is the presence of immune tolerance to the self-antigen. The present study was conducted to assess the efficacy of two previously selected anti-Id (Ab2) human scFv fragments [21] to generate an active anti-HER2 immune response. We show that vaccination with anti-Id scFv40 and scFv69 might represent a potential new therapeutic approach for the treatment of patients with HER2-positive tumors. We also demonstrate that the *in vivo *anti-tumor effects of the anti-Id scFv69 vaccine are associated with a robust anti-HER2 humoral response through a Th2-dependent mechanism.

First, we demonstrate that sera from scFv40- or scFv69-immunized BALB/c mice contain Ab1' antibodies that strongly inhibit growth of SK-OV-3 cells, suggesting a "trastuzumab-like" biological effect as reported with the *in vitro *use of the humanized anti-HER2 trastuzumab (Herceptin^®^, ROCHE, Basel, Switzerland) [24]. When adoptively transferred in nude mice bearing SK-OV-3 Luc tumor cells, these sera efficiently inhibited tumor growth. These results provide proof of the therapeutic potential of Ab1' antibodies. They also demonstrate the implication of the humoral response in the biological effects.

Second, we show that prophylactic vaccination of both virgin or primiparous MMTV.f.huHER2(Fo5) females with anti-Id scFv69 induces anti-HER2 Ab1' immune response followed by inhibition of spontaneous development of palpable tumors. The study by Finkle and colleagues indicated that early treatment with mu4D5 (the murine version of trastuzumab) was of benefit in MMTV.f.huHER2(Fo5)-transgenic females at high risk of developing huHER2-positive breast tumors. Similarly, we show that mice vaccinated with scFv69 (end of treatment at six months of age) were still free of tumors at the age of 14 months and that only one out of seven mice immunized with anti-Id scFv69 developed a tumor. Whereas in the work by Finkle *et al*. an early and prolonged treatment with mu4D5 was necessary to significantly alter mammary tumor incidence and progression, in our study, four injections of scFv69 were sufficient to elicit almost 100% protection against mammary tumors. Thus, different from the passive approach (mu4D5), active immunization with a therapeutic cancer vaccine may result in a gradual and lasting response, which could abolish tumor development and induce a long-term memory response leading to protection from disease recurrence.

Third, the analysis of the humoral response induced in immunized MMTV.f.huHER2(Fo5) mice shows that both a robust anti-scFv69 (Ab3) Th1 and an anti-HER2 (Ab1') Th2 response were associated with the delay of mammary tumor onset observed in animals treated with anti-Id scFv69. Based on these results, we think that anti-Id scFv69 could efficiently be used for the long term prevention of HER2-positive tumors via its specific anti-HER2 Th2-dependent immune mechanism.

ScFv40 and scFv69 were isolated by phage display, which is a relatively new technique to identify peptides or antibodies that mimic natural epitopes, including conformational B cell epitopes [25], as it is the case for the epitope recognized by trastuzumab [26]. The sequence analysis of scFv40 and scFv69 revealed no strong homology between their CDR1, 2 and 3 regions and the regions forming the epitope recognized by trastuzumab. This finding was expected as the epitope targeted by trastuzumab is described to be discontinuous. However, regarding the antigen mimicry capacity of the scFv69 fragment, its surface characteristics should be equivalent to those of the epitope of the selecting Ab1, although their amino acid sequence may differ [27]. Similarly, the epitope mimics generated by Riemer *et al*. [28] by phage display using trastuzumab bear no sequence homology to HER2, but they are effective in mimicking the HER2 antigen. Their sequence was subsequently matched to the third loop of HER2 at the HER2/trastuzumab interface using computational methods [29].

The low sequence homology between the CDR regions of scFv40 or scFv69 and the regions forming the HER2-epitope recognized by trastuzumab [26] could explain the lack of anti-HER2 Th1 response following immunization with scFv69. Indeed, anti-Id scFv are supposed to mimic the three-dimensional rather than the primary sequence of HER2 which is in accordance with their anti-idiotypic nature. Furthermore, the lack of anti-HER2 Th1 response may be beneficial since it might balance the immunosuppressive effect of tumor-specific regulatory T cells (Treg). Depletion or blockade of Treg cells can lead to immune protection from tumor-associated antigens that are expressed as self-antigens [30]. In our study, we demonstrate that scFv69-immunization leads to a HER2-specific Th2 immune response, suggesting that this type of vaccination could be effective in cancer patients even in the presence of immunosuppressive Treg cells.

Finally, cardiomyopathy has been shown to be a side effect of trastuzumab treatment in 7% of women following treatment with first-line anthracycline therapy and in up to 28% of women when trastuzumab is used concurrently with anthracycline [31,32]. In the study by Finkle *et al*., human HER2 was reported to be expressed in heart, but no histological or clinical evidence of cardiac or any other organ toxicity were observed following treatment with mu4D5. However, these mice were not treated with other therapeutic agents, such as anthracyclines, before or during the study. In this context, it will be useful to study the effect of immunization with scFv69 on heart function, morphology and histology after treatment with anthracycline and trastuzumab as we believe that the most promising clinical application for anti-Id scFv69-based-vaccines should be as adjuvant therapy after treatment with chemotherapy and trastuzumab. Indeed, integrating a targeted vaccine therapy after induction of a major response with a monoclonal antibody (for example, trastuzumab) could be considered as another therapeutic approach [33].

## Conclusions

In conclusion, our results demonstrate the remarkable efficiency of the scFv69 antibody fragment in protecting mice from the development of HER2-positive mammary tumors through a robust humoral response via a Th2-dependent mechanism. These results allow us to believe that scFv69 could be used as an anti-idiotype-based vaccine for adjuvant therapy in patients with HER2-positive tumors either alone or in association with concomitant passive infusion of trastuzumab.

## Abbreviations

CDR: complementary determining region; CFA: complete freund adjuvant; HER2: human epidermal growth factor receptor 2; Id: Idiotype; IFA: incomplete freund adjuvant; IFN-γ: interferon gamma; IL2: interleukin 2; i.p.: intraperitoneal; PBS: phosphate-buffered saline; scFv: single chain Fragment variable.

## Competing interests

The authors declare that they have no competing interests.

## Authors' contributions

MZL and TC made substantial contributions to the acquisition of data and to analysis and interpretation of data and drafting of the manuscript. SC made contributions to acquisition of data for the experiment of adoptive transfer of immune sera from anti-Id scFv-vaccinated mice. VG and BR made substantial contributions to conception, design and realization of *in vivo *experiments. CBM performed statistical analysis. IAA made substantial contributions to transgenic mice breeding and to *in vivo *vaccination in those mice. JPP and AP have given final approval of the manuscript version to be published. INT contributed to conception and design of the study, analysis and interpretation of data, and drafting and revision of the manuscript. All authors read and approved the final manuscript.
